# Reversal of dual epigenetic repression of non-canonical Wnt-5a normalises diabetic corneal epithelial wound healing and stem cells

**DOI:** 10.1007/s00125-023-05960-1

**Published:** 2023-07-18

**Authors:** Ruchi Shah, Tanya M. Spektor, Daniel J. Weisenberger, Hui Ding, Rameshwar Patil, Cynthia Amador, Xue-Ying Song, Steven T. Chun, Jake Inzalaco, Sue Turjman, Sean Ghiam, Jiho Jeong-Kim, Sasha Tolstoff, Sabina V. Yampolsky, Onkar B. Sawant, Yaron S. Rabinowitz, Ezra Maguen, Pedram Hamrah, Clive N. Svendsen, Mehrnoosh Saghizadeh, Julia Y. Ljubimova, Andrei A. Kramerov, Alexander V. Ljubimov

**Affiliations:** 1 Biomedical Sciences, Cedars-Sinai Medical Center, Los Angeles, CA USA; 2 Board of Governors Regenerative Medicine Institute, Cedars-Sinai Medical Center, Los Angeles, CA USA; 3 Present Address: Kura Oncology, Inc., Boston, MA USA; 4 Keck School of Medicine, University of Southern California, Los Angeles, CA USA; 5 Neurosurgery, Cedars-Sinai Medical Center, Los Angeles, CA USA; 6 Present Address: Kunshan Xinyunda Biotech Co., Ltd., Kunshan, China; 7 Present Address: Department of Basic Science, Division of Cancer Science, Loma Linda University Cancer Center, Loma Linda, CA USA; 8 Applied Genomics, Computation, and Translational Core, Cedars-Sinai Medical Center, Los Angeles, CA USA; 9 University of California Los Angeles, Los Angeles, CA USA; 10 Present Address: Mount Saint Mary’s University, Los Angeles, CA USA; 11 Present Address: Sackler School of Medicine, New York State/American Program of Tel Aviv University, Tel Aviv, Israel; 12 Present Address: California Institute of Technology, Pasadena, CA USA; 13 Duke University, Durham, NC USA; 14 Center for Vision and Eye Banking Research, Eversight, Cleveland, OH USA; 15 Surgery, Cedars-Sinai Medical Center, Los Angeles, CA USA; 16 American Eye Institute, Los Angeles, CA USA; 17 Cornea Service, New England Eye Center, Tufts Medical Center, Department of Ophthalmology, Tufts University School of Medicine, Boston, MA USA; 18 David Geffen School of Medicine, University of California Los Angeles, Los Angeles, CA USA; 19 Present Address: Terasaki Institute for Biomedical Innovation, Los Angeles, CA USA

**Keywords:** Corneal stem cells, Diabetic keratopathy, DNA methylation, Epigenetics, Gene therapy, miRNA, Wnt-5a, Wnt signalling

## Abstract

**Aims/hypothesis:**

Diabetes is associated with epigenetic modifications including DNA methylation and miRNA changes. Diabetic complications in the cornea can cause persistent epithelial defects and impaired wound healing due to limbal epithelial stem cell (LESC) dysfunction. In this study, we aimed to uncover epigenetic alterations in diabetic vs non-diabetic human limbal epithelial cells (LEC) enriched in LESC and identify new diabetic markers that can be targeted for therapy to normalise corneal epithelial wound healing and stem cell expression.

**Methods:**

Human LEC were isolated, or organ-cultured corneas were obtained, from autopsy eyes from non-diabetic (59.87±20.89 years) and diabetic (71.93±9.29 years) donors. The groups were not statistically different in age. DNA was extracted from LEC for methylation analysis using Illumina Infinium 850K MethylationEPIC BeadChip and protein was extracted for Wnt phospho array analysis. Wound healing was studied using a scratch assay in LEC or 1-heptanol wounds in organ-cultured corneas. Organ-cultured corneas and LEC were transfected with *WNT5A* siRNA, miR-203a mimic or miR-203a inhibitor or were treated with recombinant Wnt-5a (200 ng/ml), DNA methylation inhibitor zebularine (1–20 µmol/l) or biodegradable nanobioconjugates (NBCs) based on polymalic acid scaffold containing antisense oligonucleotide (AON) to miR-203a or a control scrambled AON (15–20 µmol/l).

**Results:**

There was significant differential DNA methylation between diabetic and non-diabetic LEC. *WNT5A* promoter was hypermethylated in diabetic LEC accompanied with markedly decreased Wnt-5a protein. Treatment of diabetic LEC and organ-cultured corneas with exogenous Wnt-5a accelerated wound healing by 1.4-fold (*p*<0.05) and 37% (*p*<0.05), respectively, and increased LESC and diabetic marker expression. Wnt-5a treatment in diabetic LEC increased the phosphorylation of members of the Ca^2+^-dependent non-canonical pathway (phospholipase Cγ1 and protein kinase Cβ; by 1.15-fold [*p*<0.05] and 1.36-fold [*p*<0.05], respectively). In diabetic LEC, zebularine treatment increased the levels of Wnt-5a by 1.37-fold (*p*<0.01)and stimulated wound healing in a dose-dependent manner with a 1.6-fold (p<0.01) increase by 24 h. Moreover, zebularine also improved wound healing by 30% (*p*<0.01) in diabetic organ-cultured corneas and increased LESC and diabetic marker expression. Transfection of these cells with *WNT5A* siRNA abrogated wound healing stimulation by zebularine, suggesting that its effect was primarily due to inhibition of *WNT5A* hypermethylation. Treatment of diabetic LEC and organ-cultured corneas with NBC enhanced wound healing by 1.4-fold (*p*<0.01) and 23.3% (*p*<0.05), respectively, with increased expression of LESC and diabetic markers.

**Conclusions/interpretation:**

We provide the first account of epigenetic changes in diabetic corneas including dual inhibition of *WNT5A* by DNA methylation and miRNA action. Overall, Wnt-5a is a new corneal epithelial wound healing stimulator that can be targeted to improve wound healing and stem cells in the diabetic cornea.

**Data availability:**

The DNA methylation dataset is available from the public GEO repository under accession no. GSE229328 (https://www.ncbi.nlm.nih.gov/geo/query/acc.cgi?acc=GSE229328).

**Graphical Abstract:**

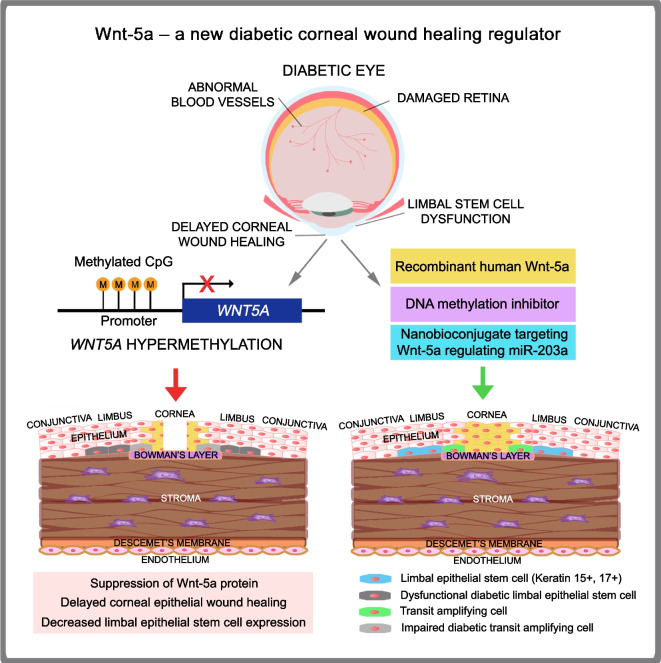

**Supplementary Information:**

The online version contains peer-reviewed but unedited supplementary material available at 10.1007/s00125-023-05960-1.


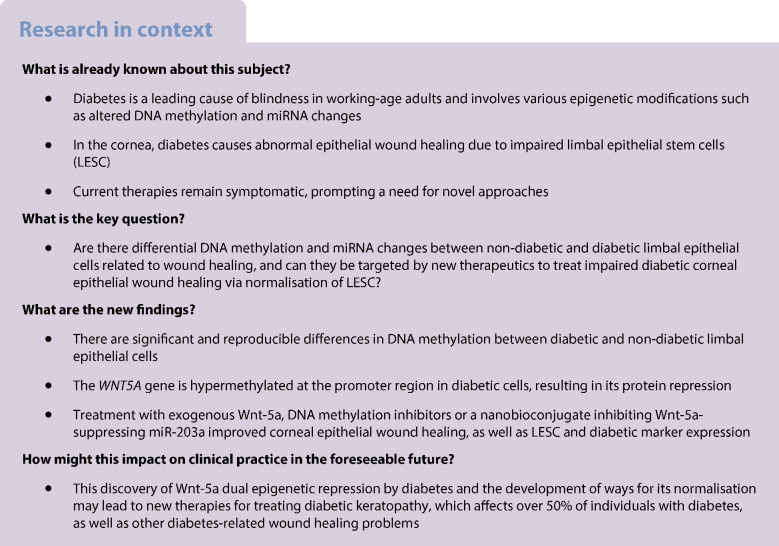


## Introduction

Ocular complications of diabetes mellitus, such as retinopathy, keratopathy, neuropathy, cataract and glaucoma, are leading causes of blindness [1, 2]. Vision loss due to diabetes mainly stems from retinal changes but up to 70% of diabetic individuals suffer from corneal problems, including keratopathy and neuropathy [3–6]. Diabetic keratopathy is characterised by impaired epithelial wound healing, barrier function and tear production, epithelial erosions and keratitis [3]. Aberrant wound healing may result from lack or dysfunction of limbal epithelial stem cells (LESC), manifested by downregulation of putative LESC markers such as keratins 15 and 17 (K15, K17), and from decreased expression of basement membrane and adhesive proteins laminins, nidogen-1 and integrin α3β1 [2, 3, 6]. Despite substantial progress in developing new treatments, current therapy with lubricants, steroids and antibiotics remains symptomatic [5]. There is an urgent need to understand the molecular mechanisms underlying diabetic keratopathy development and to design new therapeutic approaches.

Wnt-5a is a member of Wnt family of secreted signalling lipoproteins important in development, homeostasis, stem cell maintenance and regeneration [7–9]. This non-transforming Wnt activates β-catenin-independent non-canonical planar cell polarity (PCP) or Ca^2+^-dependent pathways. The PCP pathway regulates cell migration and polarity in development through Rho GTPases, specific cytoskeleton and cell polarity modulators, and c-Jun N-terminal kinase (JNK), which allows activation of JNK-dependent transcription factors influencing morphogenetic processes [7, 8]. The Ca^2+^-dependent pathway activates phospholipase C (PLC), leading to Ca^2+^ mobilisation and activation of Ca^2+^-sensing enzymes (e.g., protein kinase C [PKC]), which mediate cell migration and axon guidance [8]. Wnt-5a is expressed in corneal epithelial cells and endothelial cells [9–11]. It is increased in aniridia-related keratopathy and after TGF-α overexpression, leading to iridocorneal syndrome [12, 13]. In *Aspergillus* keratitis, increased Wnt-5a is associated with immune response [14]. In corneal endothelium, it can be induced by interleukin (IL)-1β and enhances cell migration via Cdc42 and RhoA [10]. However, there are no studies of Wnt-5a in systemic corneal diseases such as diabetes.

The existence of diabetes-related epigenetic changes [15] prompted us to compare DNA methylation patterns of diabetic and non-diabetic primary cultured human limbal epithelial cells (LEC). The goal was to identify new diabetes-altered wound healing-related protein markers that can be targeted for therapy. This study revealed Wnt-5a as a promising candidate. In this work, we used validated human organ-cultured corneas and LEC, as animal models do not reproduce all stages of human ocular diabetes [16, 17].

## Methods

### Human specimens

Human donor autopsy globes and corneas (*n*=53, electronic supplementary material [ESM] Table 1) were obtained from the National Disease Research Interchange (NDRI, Philadelphia, PA, USA) or from the Center for Vision and Eye Banking Research, Eversight (Cleveland, OH, USA) in Optisol medium (Chiron Vision, Claremont, CA, USA) within 48 h after death. The donor tissues were not statistically different in age between the two groups (23 non-diabetic: 59.87±20.89 years; 30 diabetic: 71.93±9.29 years, *p*=0.0687 by Mann–Whitney test). Of all tissues, 32 (60.4%) were from male donors and 21 (39.6%) were from female donors. Of the non-diabetic and diabetic tissues, 14 (60.9%) and 18 (60%), respectively, were from male donors and 9 (39.1%) and 12 (40%), respectively, were from female donors (ESM Table 1). All tissues, including globes, corneas and discarded donor corneoscleral rims (supplied by E. Maguen and Y. S. Rabinowitz), were used under the approved Cedars-Sinai Medical Center Institutional Review Board protocol Pro00019393.

### LEC isolation and maintenance

Primary LEC were dissociated from non-diabetic and diabetic corneas or limbal rims by dispase II digestion and cells were grown and characterised as previously described [17].

### Maintenance of human organ-cultured corneas

Corneas were organ-cultured on agar-collagen gel in serum-free medium with insulin-transferrin-selenite supplement as previously described [17].

### Genome-wide DNA methylation profiling

DNA was extracted from LESC-enriched LEC isolated from six non-diabetic donors and five diabetic donors. Bisulphite conversion was carried out according to manufacturer’s instructions (Qiagen, Valencia, CA, USA). DNA methylation was examined using Illumina Infinium 850K MethylationEPIC BeadChip covering 485,000 methylation sites and over 350,000 additional enhancer sites (Illumina, San Diego, CA, USA) as described [18].

### Bioinformatics analysis

From the array raw data, 385,642 probes were selected. Probes corresponding to X and Y chromosomes and those containing a SNP or a repetitive element within 5 bp of targeted CpG sites were excluded. Analysis was focused on the promoter region 1500 bp upstream of the transcription start site. A cut-off of 0.2 was considered optimal to define differential DNA methylation [18]. A two-way hierarchical clustering using Euclidean distance and average linkage was used for visualisation. β values were between 0 and 1, with 0 being unmethylated and 1 fully methylated. All computations and statistical analyses were performed using R 2.15.0 (R Project for Statistical Computing, Vienna, Austria) and Bioconductor 2.12 (Fred Hutchinson Cancer Research Center, Seattle, WA, USA).

### Treatment and transfection

Primary LEC were treated with human recombinant Wnt-5a (50, 100, 200 ng/ml; R&D Biosystems, Minneapolis, MN, USA) or DNA methylation inhibitors (zebularine [1, 5, 10 or 20 μmol/l, dissolved in DMSO and used at final DMSO concentrations of 0.01–0.2% vol./vol.] and decitabine [5-aza-2'-deoxycytidine 1, 5 or 10 μmol/l, dissolved in DMSO and used at final DMSO concentrations of 0.01–0.1% vol./vol.]; MilliporeSigma, St Louis, MO, USA), or canonical Wnt activator and glycogen synthase kinase (GSK)-3β inhibitor CHIR99021 (5 μmol/l, dissolved in DMSO and used at final DMSO concentration of 0.05% vol./vol.) (STEMCELL Technologies, Vancouver, BC, Canada). When LEC cultures were 60–80% confluent, they were transfected with 50 nmol/l of *WNT5A* or negative control siRNAs (Dharmacon, Lafayette, CO, USA), or hsa-miR-203a mimic and inhibitor (Thermo Fisher Scientific, Carlsbad, CA, USA) with their respective mimic or inhibitor controls, using Lipofectamine RNAiMAX (Thermo Fisher Scientific). The cells were used after 72 h. For each pair of organ-cultured corneas from the same donor, one was treated with a specific agent and the other with a control solution. Treatments included 200 ng/ml Wnt-5a with BSA as control, 20 μmol/l zebularine with DMSO as control, and nanobioconjugate (NBC) blocking miR-203a with Morpholino antisense oligonucleotide (AON) with NBC containing scrambled AON as control.

### Synthesis and characterisation of nanobioconjugates

AON against miR-203a (5′-CTAGTGGTCCTAAACATTTCAC-3′) and a negative control AON (5′-CCTCTTACCTCAGTTACAATTTATA-3′) were designed by GeneTools (Philomath, OR, USA). NBCs were synthesised in a hierarchical way as described, with quality controls at each step [19]. The characterisation and purification of NBCs is presented in ESM Table 2 and ESM Fig. 1, respectively*.* The final products were 85 kDa in molecular mass, with ζ-potential of −11.8 to −12.7 mV. Homogeneity of NBCs was verified by size-exclusion HPLC (ESM Fig. 1).

Diabetic cultured LEC and organ-cultured corneas were treated with control and therapeutic NBCs twice for 48 h with medium change (96 h total) at 15–20 μmol/l AON, then used for further analyses. NBC penetration to corneal epithelial cells was facilitated by Lipofectamine STEM transduction enhancer (Thermo Fisher).

### Western blot

Protein was extracted from LEC and homogenised limbal tissue and western blotting was performed [20]. Gel loading was normalised by β-actin. Primary antibodies are listed in ESM Table 3. IRDye 800CW or 680RD goat anti-rabbit or anti-mouse secondary antibodies (1:15,000 diluted in Tris-buffered saline with 0.1% Tween-20) were from LI-COR Biotechnology (Lincoln, NE, USA). Protein bands were visualised on Odyssey Clx System (LI-COR Biotechnology).

### Immunostaining

Transverse sections (5 µm thick) of ex vivo or organ-cultured corneas were fixed in 1% vol./vol. formalin or methanol for 5–10 min. LEC cultures were fixed in 10% formalin, permeabilised in 0.2–0.5% Triton X-100 (MilliporeSigma) and blocked in 5% vol./vol. normal goat serum [20]. Primary antibodies are described in ESM Table 3. AlexaFluor 488- or 594-conjugated secondary antibodies were from Thermo Fisher. All antibodies were diluted in PBS. Slides were mounted using ProLong Gold Antifade mounting medium with DAPI (Thermo Fisher). For each marker, the same exposure time was used when photographing stained sections or cells. Negative controls without a primary antibody were routinely included. Changes in marker expression in individual cases of organ-cultured corneas are indicated in ESM Table 4.

### LEC wound healing

Scratch wound healing assay was performed as described [20] in confluent LEC cultures, and wound closure was monitored over 24 h (48 h for NBC) using Incucyte live cell analysis system (Essen Bioscience, Ann Arbor, MI, USA). The images were quantified using ImageJ software (NIH, Bethesda, MD, USA).

### Corneal epithelial wound healing

Diabetic corneal epithelium was debrided using 5 mm paper disks soaked in 1-heptanol (MilliporeSigma) [17]. Wound closure in treated corneas was observed independently by at least two individuals every 24 h until complete healing. Healing time was recorded [20] and corneas were frozen in liquid nitrogen or embedded in Tissue-Tek OCT compound (Sakura Finetek USA, Torrance, CA, USA) for further analysis.

### Wnt signalling phospho array analysis

Protein was extracted from cultured LEC, loaded on ELISA-based antibody arrays after biotinylation of protein samples according to manufacturer’s instructions (Full Moon Biosystems, Sunnyvale, CA, USA) and detected by Cy3-conjugated streptavidin (GE Healthcare Life Sciences, Marlborough, MA, USA).

### Statistical analysis

Statistical analysis was performed using Student’s *t* test for two-group comparison using Prism9 (GraphPad Software, San Diego, CA, USA), with *p*<0.05 considered significant. Donor ages in non-diabetic and diabetic groups were compared using two-tailed Mann–Whitney test. Normality was confirmed by Shapiro–Wilk test. Each cell culture experiment was performed in triplicate from three to six different donors, and a minimum of four individual corneal pairs were used for each treatment. Values were expressed as mean ± SEM, except for age analysis (mean ± SD).

## Results

### Diabetic and non-diabetic cultured LEC have differential DNA methylation profiles

**Fig. 1:**
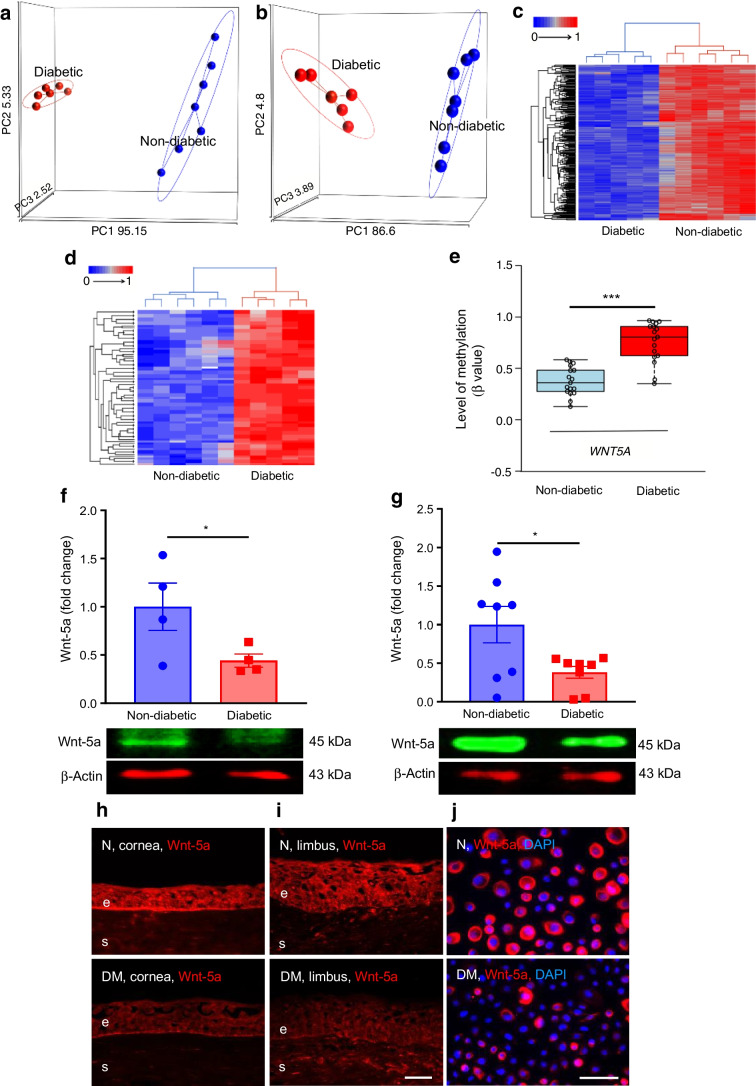
*WNT5A* gene is hypermethylated and has reduced expression in diabetic vs non-diabetic LEC enriched in LESC. (**a**, **b**) PCA plot of gene clusters showed clear segregation between diabetic and non-diabetic DNA hypomethylation patterns (**a**) and hypermethylation patterns (**b**). (**c**, **d**) Hierarchical clustering of 364 hypomethylated probes (**c**) and 82 hypermethylated probes (**d**) in primary diabetic (*n*=5) vs non-diabetic (*n*=6) LEC. (**e**) Beta values of DNA methylation levels in probes corresponding to 1000 bp across the transcription start site of *WNT5A* gene at two promoter sites in non-diabetic and diabetic LEC. Each circle represents a probe, and line in box indicates median of methylation level of *WNT5A*. Non-diabetic and diabetic donors did not differ statistically from each other by age (54.20±29.40 years for non-diabetic vs 72.60±16.16 years for diabetic donors, *p*=0.3095 by Mann–Whitney test). (**f**, **g**) Decreased Wnt-5a protein levels in diabetic vs non-diabetic ex vivo limbal tissue (*n*=4) (**f**) and LEC (*n*=8) (**g**) were revealed by western blot analysis. (**h**–**j**) Wnt-5a immunostaining showed marked decrease in diabetic samples, similar to western blot analysis, in ex vivo corneal (**h**) and limbal sections (*n*=3) (**i**), and cultured LEC (*n*=3) (**j**). All values are mean ± SEM. *p<0.05 and ***p<0.001 (unpaired *t* test). FDR*p*<0.05; Δβ ± 0.2. Scale bar, 20 µm. The blot cut-outs for Wnt-5a and β-actin in (**f**) and (**g**) are from the same gel in each graph. DM, diabetic; e, epithelium; N, non-diabetic; s, stroma

Global DNA methylation analysis revealed differential methylation between non-diabetic and diabetic LEC. Using a cut-off of ≥0.2 and a false discovery rate (FDR)-adjusted *p* value of <0.05, we identified a cluster of 446 probes with significant differences in DNA methylation between the groups and clear segregation by principal component analysis (PCA) plot [19] (Fig. 1a,b). Among these probes, 364 were hypomethylated (Fig. 1c) and 82 were hypermethylated (Fig. 1d) in diabetic vs non-diabetic LEC.

### DNA hypermethylation of *WNT5A* suppresses its protein levels in diabetic corneal epithelium

*WNT5A* gene promoter was significantly hypermethylated in diabetic vs non-diabetic LEC (Fig. 1e). This was accompanied by decreased Wnt-5a protein levels in diabetic cultured LEC and ex vivo corneas. Western blotting revealed that the Wnt-5a level was decreased by 56% (*p*<0.05; Fig. 1f) and 65% (*p*<0.05; Fig. 1g) in diabetic limbus and LEC, respectively, vs non-diabetic controls. Immunostaining also showed that Wnt-5a was decreased in diabetic central cornea (Fig. 1h), limbus (Fig. 1i) and cultured LEC (Fig. 1j). Similarly, Wnt-5a level was decreased by 60% (*p*<0.05) in diabetic vs non-diabetic LEC by ELISA (Full Moon Biosystems), definitively confirming suppression of Wnt-5a in diabetes. These changes appeared specific to Wnt-5a, as levels of a typical canonical Wnt-3a (ESM Fig. 2a, b) and a non-canonical Wnt-11 (ESM Fig. 2c, d) remained unchanged between the groups.

### Wnt-5a improves wound healing and limbal stem cell phenotype in diabetic LEC and organ-cultured corneas

**Fig. 2:**
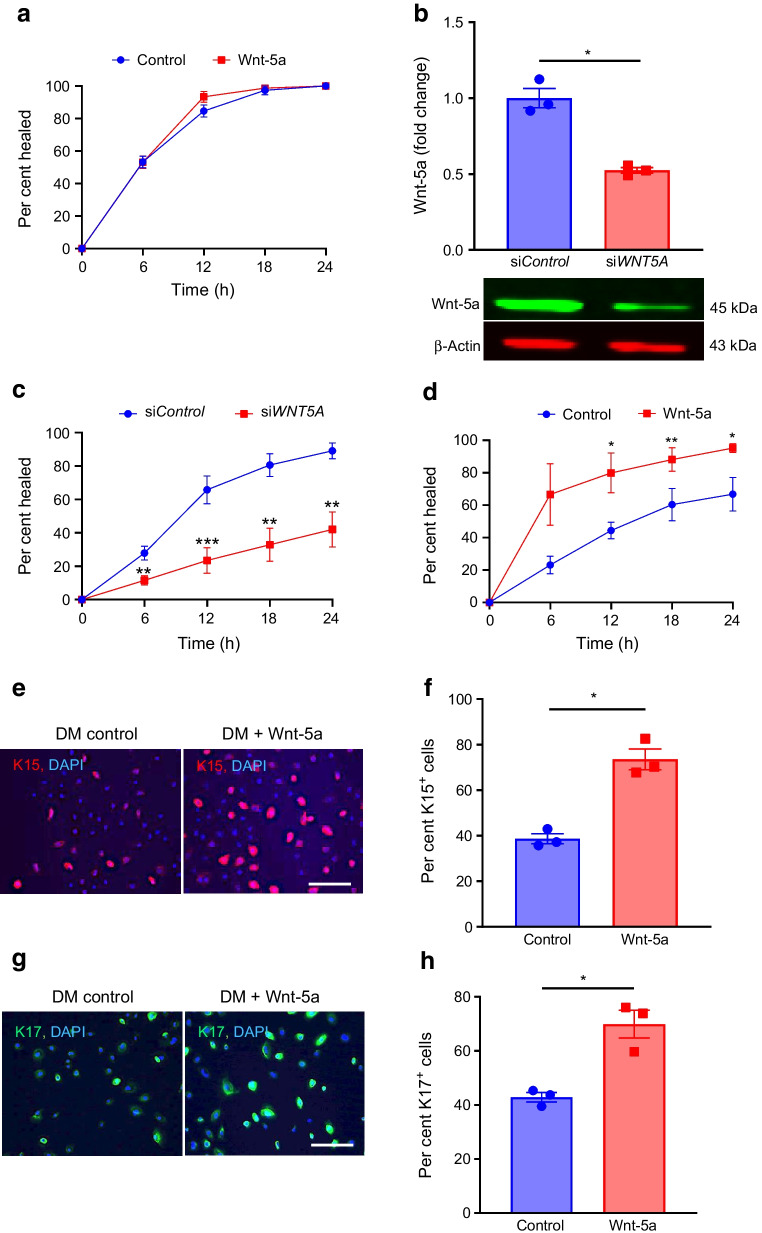
Wnt-5a improves wound healing and limbal stem cell phenotype in diabetic but not in non-diabetic LEC. (**a**) Scratch wound healing dynamics in non-diabetic (*n*=3) cultured LEC was similar with or without Wnt-5a (200 ng/ml). (**b**, **c**) Transfection of non-diabetic LEC with *WNT5A* siRNA showed decreased expression of Wnt-5a (*n*=3) (**b**) and significantly impaired wound healing (*n*=4) (**c**). (**d**–**h**) Addition of Wnt-5a (200 ng/ml) to diabetic LEC (*n*=4) significantly enhanced wound healing (**d**) and markedly increased immunostaining of stem cell markers K15 (**e**, **f**) and K17 (**g**, **h**) (*n*=3). Values are mean ± SEM. *p<0.05, **p<0.01 and ***p<0.001 vs control at the same time point. Paired *t* test. The blot cut-outs for Wnt-5a and β-actin are from the same gel in (**b**). DM, diabetic. Scale bar, 20 µm

**Fig. 3:**
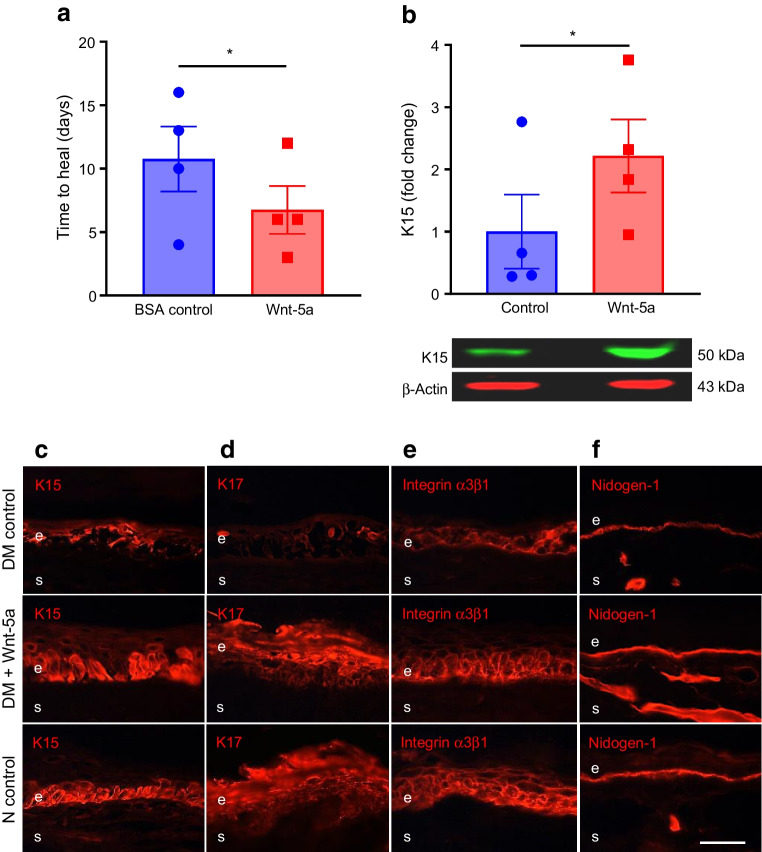
Wnt-5a improves wound healing and limbal stem cell phenotype in diabetic organ-cultured corneas. (**a**) Wound healing time in 1-heptanol-wounded diabetic corneas was significantly decreased after treatment with Wnt-5a protein (200 ng/ml) vs BSA (control) (*n*=4). (**b**) Western blot analysis showed that Wnt-5a treatment significantly increased the levels of putative limbal stem cell marker K15 (*n*=4). (**c**–**f**) Corneal section immunostaining revealed that Wnt-5a treatment normalised the expression of putative LESC markers K15 (**c**) and K17 (**d**) as well as the expression of diabetic markers integrin α3β1 (**e**) and nidogen-1 (**f**) in healed corneas (*n*=4). Normal (non-diabetic) ex vivo patterns of these markers are also shown for comparison. Values are mean ± SEM. *p<0.05 vs control (paired *t* test). The blot cut-outs for K15 and β-actin are from the same gel in (**b**). Scale bar, 20 µm. DM, diabetic; e, epithelium; N, non-diabetic, s, stroma

As Wnt-5a plays a critical role in stem cell maintenance and regeneration [7, 9], we assessed the effects of reduced Wnt-5a levels on wound healing and LESC markers. In a scratch wound healing assay, non-diabetic LEC treated with exogenous Wnt-5a (200 ng/ml; dose established in preliminary experiments, ESM Fig. 3) revealed no significant difference in wound closure over 24 h when compared with untreated controls (Fig. 2a). To test whether this lack of effect was due to high Wnt-5a content, cells were treated with a *WNT5A* siRNA cocktail that reduced the Wnt-5a level by 50% vs control siRNA (*p*<0.05; Fig. 2b). Indeed, transfection of non-diabetic LEC with *WNT5A* siRNA significantly delayed wound healing by 53.3% at 24 h (*p*<0.01; Fig. 2c). Concomitantly, Wnt-5a treatment of diabetic LEC resulted in a 1.4-fold increase in wound healing vs untreated controls by 24 h (*p*<0.05; Fig. 2d) and showed increased levels of putative LESC markers K15 (Fig. 2e,f; about 1.9-fold more K15^+^ cells vs control, *p*<0.05) and K17 (Fig. 2g,h; about 1.6-fold more K17^+^ cells vs control, *p*<0.05). Similarly, Wnt-5a treatment of wounded diabetic organ-cultured corneas reduced healing time by 37% (*p*<0.05; Fig. 3a) vs BSA treatment (control), with acquisition of normal-like patterns of LESC markers K15 (Fig. 3b,c) and K17 (Fig. 3d), and diabetes-suppressed integrin α3β1 (Fig. 3e) and nidogen-1 (Fig. 3f). Furthermore, the immunostaining for phosphorylated/activated wound healing mediators, p-Akt (ESM Fig. 4a) and p-p38 (ESM Fig. 4b), was increased in Wnt-5a-treated corneas vs BSA-treated control corneas. Ki-67 immunostaining showed that Wnt-5a treatment appeared to have no effect on cell proliferation (ESM Fig. 5a) and did not cause apoptosis, as inferred from activated caspase-3 immunostaining (ESM Fig. 5b) vs BSA controls.

### Wnt-5a activates non-canonical Ca^2+^ signalling pathway in diabetic LEC

**Fig. 4:**
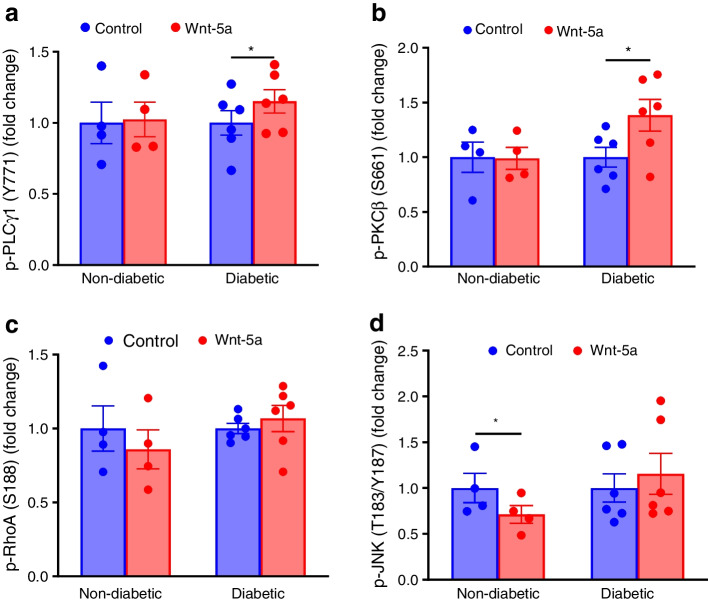
Wnt-5a activates non-canonical Ca^2+^ signalling in diabetic LEC. Wnt phospho array analysis of phosphorylated p-PLCγ1 (Y771) (**a**), p-PKCβ (S661) (**b**), p-RhoA (S188) (**c**) and p-JNK (T183/Y187) (**d**) in non-diabetic (*n*=4) and diabetic LEC (*n*=6) with or without Wnt-5a (200 ng/ml). In diabetic LEC, Wnt-5a treatment significantly increased Ca^2+^ pathway components p-PLCγ1 and p-PKCβ but not PCP pathway components p-RhoA or p-JNK. Normal cells did not activate any of the shown signalling intermediates upon treatment. Values are mean ± SEM. *p<0.05 vs control (paired *t* test)

We next determined signalling pathways activated by Wnt-5a in human LEC. ELISA-based phospho antibody arrays revealed that Wnt-5a treatment of diabetic but not non-diabetic LEC significantly increased phosphorylation of members of the Ca^2+^-dependent non-canonical pathway, PLCγ1 at Tyr771 (Fig. 4a) and PKCβ at Ser661 (Fig. 4b) by 1.15-fold (*p*<0.05) and 1.36-fold (*p*<0.05), respectively. However, RhoA (Fig. 4c) or JNK (Fig. 4d) of the PCP pathway were not activated. Wnt-5a also activated wound healing signalling mediators, including Akt, proto-oncogene tyrosine-protein kinase Src, extracellular signal-regulated kinase (ERK)3 and 8 and TGF-β-activated kinase 1 (TAK1) (ESM Table 5). Importantly, specific canonical Wnt signalling activation by GSK-3β inhibitor CHIR99021 did not appear to accelerate wound healing of diabetic LEC, in contrast to Wnt-5a. CHIR99021 combination with Wnt-5a only slightly increased healing over Wnt-5a alone (ESM Fig. 6).

### DNA methylation inhibitors increase Wnt-5a levels and wound healing in diabetic LEC and organ-cultured corneas

**Fig. 5:**
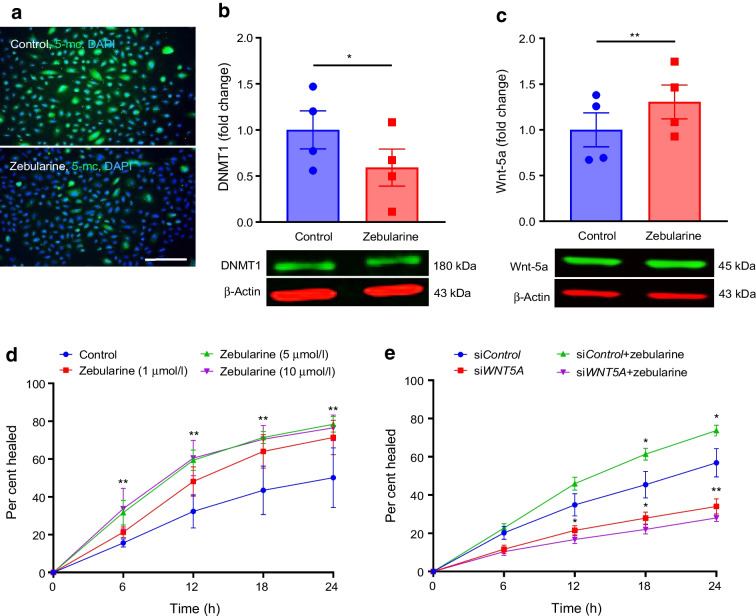
Zebularine accelerates wound healing in diabetic LEC via Wnt-5a stimulation. (**a**, **b**) Zebularine (5 μmol/l) reduced the levels of 5-methylcytosine (*n*=3) (**a**) and DNMT1 (*n*=4) (**b**) in diabetic LEC. (**c**) Wnt-5a level was significantly increased by zebularine (5 μmol/l) in diabetic LEC (*n*=4). (**d**) Zebularine dose-dependently stimulated scratch wound healing of diabetic LEC (*n*=3). (**e**) *WNT5A* siRNA abrogated the stimulating effect of zebularine on scratch wound healing in diabetic LEC (*n*=3). Values are mean ± SEM. *p<0.05 and **p<0.01 vs control at the same time point (paired *t* test). The blot cut-outs for DNMT1 and β-actin and Wnt-5a and β-actin are from the same gel in (**b**) and (**c**),respectively. Scale bar, 40 µm. 5-mc, 5-methylcytosine

After establishing the critical role of Wnt-5a in diabetic corneal wound healing, we explored ways to stimulate this process by modulating Wnt-5a expression. As *WNT5A* promoter was hypermethylated in diabetic LEC, we examined the effect of zebularine, a demethylating agent that inhibits DNA methyltransferases (mostly DNA methyltransferase 1 [DNMT1]) [21], on diabetic corneal epithelial wound healing. Diabetic LEC treated with 5 µmol/l zebularine showed substantially decreased 5-methylcytosine (Fig. 5a) and significantly (40.9%) reduced DNMT1 levels (*p*<0.05; Fig. 5b), like the DNMT1 depletion reported previously for human bladder cancer cells [21], indicating effective DNA demethylation. Zebularine treatment increased Wnt-5a levels by 1.37-fold (*p*<0.01; Fig. 5c), leading to a dose-dependent improvement in wound healing over 24 h with a 1.6-fold (*p*<0.01) increase vs untreated control (Fig. 5d). This was confirmed with an FDA-approved demethylating agent (decitabine [5-aza-2′-deoxycytidine]), which resulted in a similar dose-dependent increase in wound healing (ESM Fig. 7). Decitabine was slightly less active than zebularine in wound healing stimulation and is reportedly more toxic and unstable in aqueous solutions [21]. Hence, subsequent experiments were performed with zebularine. Diabetic LEC transfection with *WNT5A* siRNA abrogated zebularine stimulation of wound healing (*p*<0.05; Fig. 5e) by 24 h, suggesting that the effect of zebularine was primarily due to the inhibition of *WNT5A* promoter DNA methylation.

**Fig. 6:**
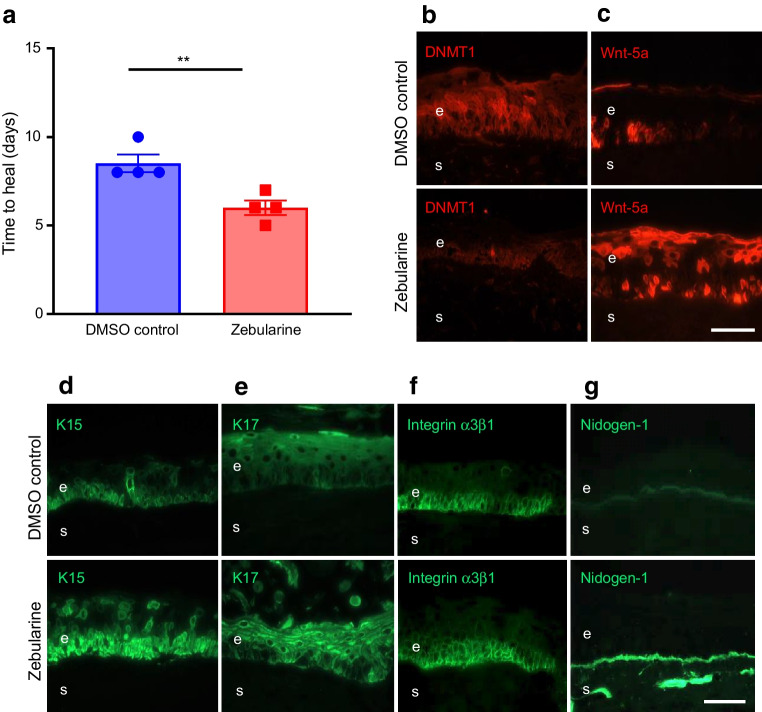
Zebularine improves wound healing and limbal stem cell phenotype in diabetic organ-cultured corneas via Wnt-5a. (**a**) Wound healing time was significantly decreased in heptanol-wounded diabetic corneas treated with zebularine (20 µmol/l) vs DMSO (control) (*n*=4). (**b**–**g**) Immunostaining (*n*=3) showed that zebularine decreased DNMT1 (**b**) but increased Wnt-5a (**c**) levels in healed corneas. Zebularine also increased limbal levels of putative stem cell markers, K15 (**d**) and K17 (**e**), as well as diabetic markers integrin α3β1 (**f**) and nidogen-1 (**g**). Values are mean ± SEM. **p<0.01 vs control (paired *t* test). Scale bar, 20 µm. e, epithelium; s, stroma

In diabetic organ-cultured corneas, treatment with 20 µmol/l zebularine significantly decreased corneal epithelial wound healing time vs DMSO control (30% stimulation, *p*<0.01; Fig. 6a), decreased DNMT1 levels (Fig. 6b) and increased Wnt-5a levels (Fig. 6c). Zebularine markedly increased the expression of K15 (Fig. 6d), K17 (Fig. 6e), integrin α3β1 (Fig. 6f) and nidogen-1 (Fig. 6g), and wound healing mediators, p-Akt (ESM Fig. 4c), p-p38 (ESM Fig. 4d), and p-ERK1/2 (ESM Fig. 4e). Zebularine did not induce apoptosis or affect cell proliferation vs DMSO control (ESM Fig. 5).

### Blocking of Wnt-5a-inhibiting miR-203a increases Wnt-5a levels and wound healing in diabetic LEC and organ-cultured corneas

Since miRNAs regulate post-transcriptional gene expression [22], we also considered miRNAs affecting Wnt-5a and their changes in the diabetic cornea. Our previous miRNA-seq study revealed *WNT5A*-interacting miR-203a among several other *WNT5A*-interacting miRNAs, with close to two fold upregulation in diabetic vs non-diabetic ex vivo limbal cells (complete data were uploaded to public GEO repository under accession GSE97069). Diabetic LEC transfected with miR-203a inhibitor showed increased Wnt-5a levels, suggesting that miR-203a regulated Wnt-5a expression (ESM Fig. 8a). Moreover, miR-203a did not affect levels of LESC marker ΔNp63 in diabetic LEC (ESM Fig. 8b), contrary to its effect in skin keratinocytes [23].

**Fig. 7:**
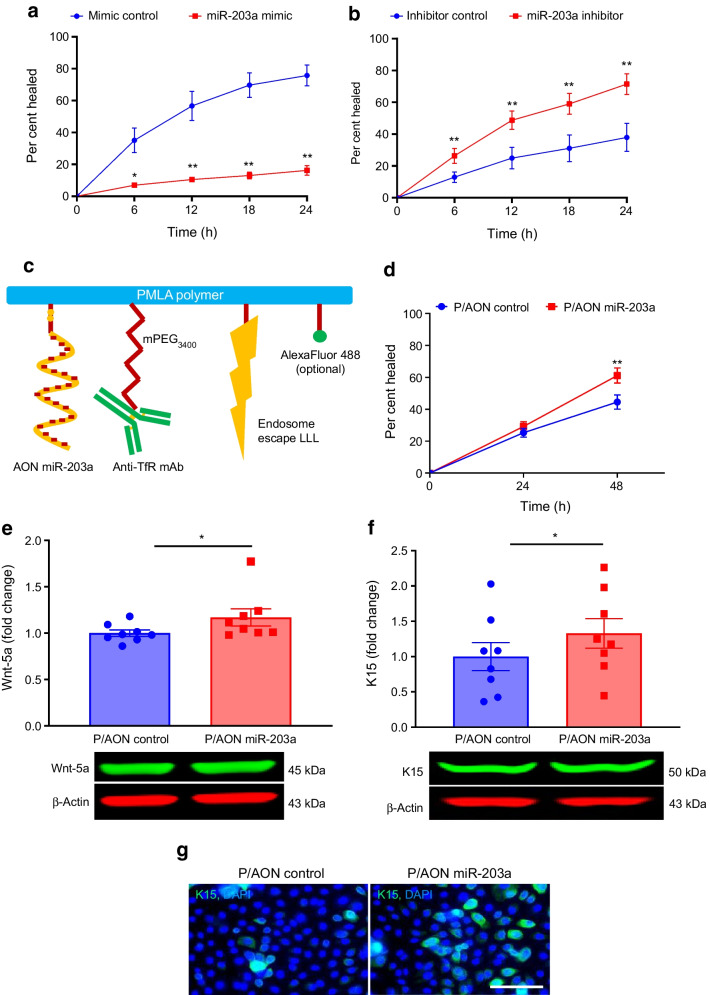
Inhibition of Wnt-5a-regulating miR-203a accelerates wound healing in diabetic LEC. (**a**) Transfection with miR-203a mimic significantly inhibited wound healing in non-diabetic LEC (*n*=3). (**b**) Wound healing in diabetic LEC transfected with miR-203a inhibitor was significantly stimulated (*n*=4). (**c**) Schematic structure of PMLA-based biodegradable NBC containing AON targeting miR-203a (detailed in the Methods). (**d**–**g**) Treatment of diabetic LEC (*n*=8) with this NBC stimulated wound healing (**d**) and increased the protein levels of Wnt-5a (**e**) and K15 (**f**), as shown by western blot, and of K15, as shown by immunostaining (**g**), compared with NBC with control scrambled AON. Values are mean ± SEM. *p<0.05 and **p<0.01 vs control at the same time point (paired *t* test). The blot cut-outs for Wnt-5a and β-actin and K15 and β-actin are from the same gel in (**e**) and (**f**), respectively. Scale bar, 20 µm. Anti-TfR mAb, cell-targeting monoclonal antibody to transferrin receptor; AON, morpholino antisense oligonucleotide; LLL, trileucine endosome escape unit; P/AON, polymalic acid with covalently attached antisense oligonucleotide

Non-diabetic LEC transfection with a miR-203a mimic to reduce Wnt-5a caused a significant 78% decrease in area healed by 24 h (*p*<0.01) vs mimic control (Fig. 7a). Conversely, diabetic LEC transfected with specific miR-203a inhibitor (to increase Wnt-5a) showed a 1.85-fold increase (*p*<0.01) in healed area by 24 h vs inhibitor control (Fig. 7b).

**Fig. 8:**
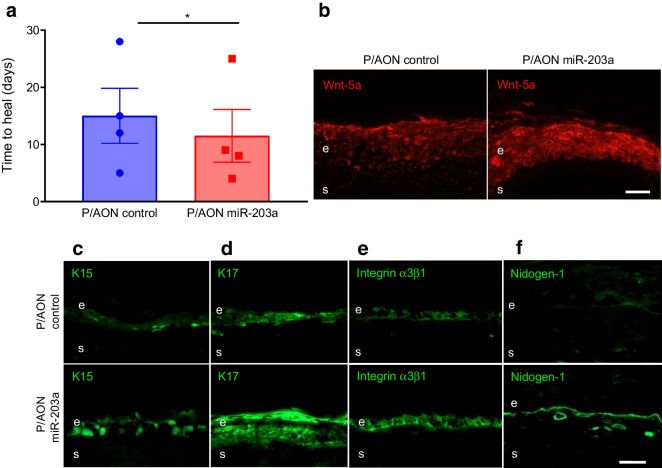
NBC with AON suppressing miR-203a improves wound healing and limbal stem cell phenotype in diabetic corneal organ cultures via Wnt-5a. (**a**) Wound healing time in heptanol-wounded diabetic corneas treated with P/AON miR-203a was significantly reduced vs P/AON control (*n*=4). (**b**–**f**) Immunostaining of Wnt-5a (**b**), putative limbal stem cell makers K15 (**c**) and K17 (**d**), as well as diabetic markers integrin α3β1 (**e**) and nidogen-1 (**f**), in healed corneas (*n*=4) showed marked increase after NBC treatment. Values are mean ± SEM. *p<0.05 vs control (paired *t* test) Scale bar, 20 µm. AON, morpholino antisense oligonucleotide; P/AON, polymalic acid with covalently attached antisense oligonucleotide

To suppress miR-203a in diabetic organ-cultured corneas, we designed a novel NBC carrying AON against miR-203a. This NBC was engineered on natural, biodegradable and non-toxic polymalic acid (PMLA) scaffold with covalently attached functional moieties including AON [19], a monoclonal antibody to transferrin receptor (TfR) for receptor-mediated endocytosis, a trileucine moiety for endosomal escape at low pH, and a tracking fluorescent dye (Fig. 7c) [19]. Diabetic and non-diabetic LEC abundantly express TfR mediating NBC uptake [19]. Diabetic LEC cultured for 4 days with this NBC at 15 µmol/l AON showed accelerated scratch wound healing by 1.4-fold (*p*<0.01; Fig. 7d) vs control NBC at 48 h and showed modest yet significant increases in Wnt-5a levels (by 1.17-fold, *p*<0.05; Fig. 7e) and K15 levels (by 1.3-fold, *p*<0.05; Fig. 7f,g). Similarly, when compared with control NBC treatment, treatment of diabetic organ-cultured corneas with 20 µmol/l of the therapeutic NBC enhanced corneal wound healing (by 23.3%, *p*<0.05; Fig. 8a) and increased levels of Wnt-5a (Fig. 8b), LESC markers K15 (Fig. 8c) and K17 (Fig. 8d), diabetic markers integrin α3β1 (Fig. 8e) and nidogen-1 (Fig. 8f), and wound healing mediators p-Akt (ESM Fig. 4f), p-p38 (ESM Fig. 4g) and p-ERK1/2 (ESM Fig. 4h). Similar to zebularine, the therapeutic NBC did not cause apoptosis or affect cell proliferation (ESM Fig. 5).

## Discussion

Genetic and environmental factors play important roles in the development of diabetes [15]. Epigenetic changes altering gene and protein expression through DNA methylation, histone modifications and non-coding miRNA are widely associated with ocular diseases [20, 24]. Age-related macular degeneration, retinoblastoma, cataract, pterygium, diabetic retinopathy and glaucoma have been linked to altered DNA methylation and histone acetylation [25–29]. Several miRNAs have been implicated in ocular disorders such as retinitis pigmentosa, age-related cataract, age-related macular degeneration, corneal dystrophy, autoimmune uveoretinitis, pathological angiogenesis, diabetic retinopathy and ocular neoplasms [28, 30].

To our knowledge, this is the first study that compared global DNA methylation profiles of diabetic and non-diabetic LEC enriched in LESC and found marked differences between these cells. Hypermethylation of the *WNT5A* gene promoter in diabetic LEC was of significant interest due to the role of Wnt signalling in controlling cell fate, proliferation, migration, stem cell maintenance and tissue homeostasis [31]. This diabetes-associated epigenetic change translated into a dramatic reduction of Wnt-5a levels in diabetic ex vivo corneas and cultured LEC. It played a functional role in epithelial wound healing, as replenishment with recombinant Wnt-5a stimulated diabetic corneal wound healing and expression of putative LESC (K15, K17) and diabetes-suppressed (integrin α3β1 and nidogen-1) markers, alleviating important diabetic changes [3, 17] in these corneas. Additional evidence for its role was obtained in non-diabetic LEC, where wound healing was significantly slowed by siRNA-mediated Wnt-5a inhibition. Mechanistically, Wnt-5a addition to diabetic LEC activated the non-canonical Ca^2+^ signalling pathway, important for cell migration through the phosphorylation of PLCγ1 and PKCβ [8]. However, non-canonical PCP signalling (p-RhoA and p-JNK) did not appear to be activated by Wnt-5a. Wnt-5a was non-toxic and did not affect cell proliferation. Overall, Wnt-5a improved several aspects of diabetic keratopathy and may be considered for future therapy.

Activation of canonical Wnt signalling together with Wnt-5a addition only slightly increased human diabetic LEC wound healing vs Wnt-5a alone, indicating the minor role of canonical signalling in this process. However, some studies suggest that canonical Wnt/β–catenin signalling is also suppressed in diabetic mouse corneas and that its activation would accelerate wound healing [32, 33]. The differences between our study and these previous studies may be due to the following factors: (1) different type of diabetes (type 1 in mouse studies vs predominantly type 2 in ours); (2) experimental conditions (wound healing assays were performed in non-diabetic animals, whereas we used human diabetic cells); and (3) possibility of activation of other pathways by their inhibitors and activators (glucose transporters or non-canonical pathway). Nonetheless, this issue should be further examined with direct modulation of signalling pathways in diabetic models in vivo*.*

New treatment approaches for diabetic corneal disease have been investigated [3] but mainstream treatment remains symptomatic [5]. We have documented molecular alterations in the human diabetic corneal epithelium that were targeted by viral and nanogene therapy to improve wound healing and LESC marker expression [3, 6, 17, 19]. To take advantage of the beneficial effects of Wnt-5a for possible therapy, we attempted to increase its expression in diabetic corneas using DNA demethylating agents, in particular zebularine, a stable anticancer demethylating agent with low toxicity [21, 34]. Zebularine induces minimal side effects in mice, even with prolonged treatment [34], and can activate wound healing during mouse ear pinna regeneration [35]. Zebularine is a DNMT inhibitor [36], as are two other chemically labile and more toxic demethylating agents, 5-azacytidine (Vidaza) and decitabine (Dacogen), which are FDA-approved anticancer drugs [37–39]. Zebularine was non-toxic up to 20 μmol/l in diabetic organ-cultured corneas, as indicated by lack of increased active caspase-3 immunostaining. As anticipated, zebularine increased Wnt-5a expression and efficiently promoted LEC wound healing as compared with the less-stable 5-azacytidine, which was toxic above 1 μmol/l (data not shown). The FDA-approved decitabine demonstrated a similar but less pronounced effect on diabetic LEC wound healing compared with zebularine.

Zebularine might affect methylation of multiple genes including wound healing mediators [40]. We found that the promotion of diabetic corneal wound healing by zebularine was likely due to the inhibition of *WNT5A* methylation and restoration of Wnt-5a levels, as transfection with *WNT5A* siRNA abrogated this effect. Such selectivity may be due to a relatively low dose (20 µmol/l of zebularine vs 100–1000 µmol/l used by others) [21, 41]. This specificity is important for future therapeutic development using topical zebularine for impaired diabetic corneal wound healing, which would not require long-term treatment.

Genome-wide analysis has documented differential miRNA signatures in diabetic vs non-diabetic human limbus [42]. This analysis showed that Wnt-5a-inhibiting miR-203a was overexpressed in diabetic vs non-diabetic LEC. Functional experiments revealed that miR-203a decreased wound healing in non-diabetic LEC whereas its inhibitor increased wound healing in diabetic LEC. These changes appeared to be specific for Wnt-5a, as miR-203a inhibitor in diabetic LEC did not increase putative LESC marker ΔNp63, which is a miR-203 target in skin keratinocytes [23].

Gene therapy is a promising approach for corneal wound healing due to easy corneal accessibility for drug delivery [43]. Morpholino AONs constitute an ideal tool for such therapy as they are very stable, are sequence-specific, do not alter unrelated genes unlike siRNA, and have a long-lasting action [44]. Some AONs are already in clinical use [45–47]. We previously used adenoviral gene therapy in diabetic corneas but found that it elicited toxicity in LEC [17]. This prompted us to develop non-toxic nano therapy [48] for diabetic corneas using novel NBCs on PMLA scaffold [19]. Our NBCs (20–30 nm) are non-toxic, are biodegradable, and have covalently attached multiple functional moieties including cell-targeting antibody to TfR for active receptor-mediated endocytosis, nuclease-resistant morpholino AON inhibiting miR-203a, and a trileucine pH-dependent endosome escape unit, making them superior to conventional non-covalent and non-targeted nanoparticles [19, 43]. Treatment of diabetic LEC with NBC significantly improved wound healing and increased Wnt-5a and LESC marker expression. NBCs thus represent a promising gene therapy alternative to normalise diabetic corneal functions.

A limitation of this study is that both zebularine and NBC provided significant but partial improvement of diabetic corneal epithelial wound healing. This limitation may be circumvented in the future using a combination of these agents. As they influence different Wnt-5a regulatory pathways, their combination could provide more efficient corneal normalisation.

The presented data demonstrate a novel and unique mechanism of Wnt-5a regulation in diabetic corneal cells involving dual epigenetic repression by *WNT5A* promoter methylation and miR-203a action. A similar regulation has only been described for oestrogen receptor α36 in breast cancer cells [49] but never for a systemic disease such as diabetes. Reversal of this repression in diabetic cells and corneas improved epithelial wound healing and stem cell marker expression. Wnt-5a has emerged as a new diabetic corneal marker regulating wound healing and stem cell function. This may also be significant for impaired wound healing in other diabetes complications, including diabetic foot ulcers, which share some neurovascular, sensory and immunological compromise with diabetic eye disease [50]. Novel therapies to reverse both types of epigenetic silencing could benefit corneal function and may also prove to be beneficial in other wound healing-related diabetic complications.

## Supplementary Information

Below is the link to the electronic supplementary material.

Supplementary file1 (PDF 801 KB)

## Data Availability

The DNA methylation dataset is available from the public GEO repository under accession no. GSE229328 (https://www.ncbi.nlm.nih.gov/geo/query/acc.cgi?acc=GSE229328).

## Abbreviations

AON: Antisense oligonucleotide
DNMT1: DNA methyltransferase 1
FDR: False discovery rate
GSK-3β: Glycogen synthase kinase-3 beta
JNK: c-Jun N-terminal kinase
K15: Keratin 15
K17: Keratin 17
LEC: Limbal epithelial cells
LESC: Limbal epithelial stem cells
NBC: Nanobioconjugate
PCA: Principal component analysis
PCP: Planar cell polarity
PKC: Protein kinase C
PLC: Phospholipase C
PMLA: Polymalic acid
TfR: Transferrin receptor
