# Supplementary material for: Reversal of dual epigenetic repression of non-canonical Wnt-5a normalises diabetic corneal epithelial wound healing and stem cells
Source: Diabetologia. 2023 Jul 18;66(10):1943–58. doi: 10.1007/s00125-023-05960-1 (PMC10474199; doi:10.1007/s00125-023-05960-1)
Supplement: Supplementary file 1 — Supplementary file1 (PDF 801 KB) [file 125_2023_5960_MOESM1_ESM.pdf]

Electronic Supplementary Materials (ESM)

ESM Table 1. Non-diabetic and diabetic donor characteristics

Case #	Age (years)	Sex	DM Type	Duration (years)	Cause of Death	DM medications	Used for
N09-18	75	M	N/A	N/A	Respiratory failure	N/A	Organ culture
N10-08-1	62	M	N/A	N/A	Cardiac arrest	N/A	DNA methylation
N10-18	67	M	N/A	N/A	Stroke	N/A	*Ex vivo*
N10-21	79	M	N/A	N/A	Stroke	N/A	*Ex vivo*
N13-01	78	F	N/A	N/A	Heart disease	N/A	LEC culture
N13-21	20	M	N/A	N/A	Asphyxiation	N/A	LEC culture, DNA methylation
N13-38-1	27	M	N/A	N/A	Respiratory failure	N/A	LEC culture, DNA methylation
N14-01	86	F	N/A	N/A	COPD	N/A	LEC culture
N14-02	65	M	N/A	N/A	Unknown	N/A	LEC culture
N16-12	86	F	N/A	N/A	Stroke / CVA	N/A	LEC culture, DNA methylation
N16-14	76	F	N/A	N/A	Cardiac/pulmonary failure	N/A	LEC culture, DNA methylation
N16-18	64	M	N/A	N/A	Lung cancer	N/A	LEC culture
N17-32	36	F	N/A	N/A	Metastatic cardiac angiosarcoma	N/A	LEC culture
N17-46	23	M	N/A	N/A	MBF injuries to head and neck 2nd to MVA	N/A	LEC culture
N18-20	70	M	N/A	N/A	Respiratory failure	N/A	*Ex vivo*
N18-26	73	M	N/A	N/A	Myocardial infarction	N/A	*Ex vivo*
N18-28	32	M	N/A	N/A	Ligature hanging	N/A	LEC culture
N18-32	31	M	N/A	N/A	Multiple vehicular blunt force injuries of head, neck, torso	N/A	LEC culture
N19-21	76	F	N/A	N/A	Anoxic brain injury post code	N/A	LEC culture
N19-22	66	F	N/A	N/A	Respiratory failure	N/A	LEC culture
N19-23	55	F	N/A	N/A	Myocardial infarction	N/A	LEC culture
N19-25	58	M	N/A	N/A	Metastatic lung cancer	N/A	LEC culture

N19-51	72	F	N/A	N/A	AAA dissection	N/A	Organ culture
DR13-13	78	M	NIDDM	31	Respiratory failure	Glyburide, Humalog, Lantus	LEC culture
DR10-15	70	M	NIDDM	40	Subarachnoid hemorrhage	Novalin N, Novalin R	*Ex vivo*
DR10-16	63	M	NIDDM	10	Aspiration pneumonia	Unknown	*Ex vivo*
DM13-02	91	M	NIDDM	6	Pneumonia	Unknown	LEC culture, DNA methylation
DM13-03	63	F	NIDDM	Unknown	Respiratory failure	Insulin, Glucophage, GlucaGen	LEC culture, DNA methylation
DM13-04	67	M	IDDM	13	Coronary artery disease	Lantus	LEC culture, DNA methylation
DR13-15	54	M	IDDM	46	Acute renal failure	Humalog	DNA methylation
DM14-35	62	F	NIDDM	30	Multisystem organ failure	Metformin	LEC culture
DM14-41	67	M	NIDDM	21	Possible myocardial infarction	Unknown	LEC culture
DM14-43	73	F	NIDDM	10	Congestive heart failure, acute renal failure	Unknown	LEC culture
DM15-04	81	M	NIDDM	20	Congestive heart failure	Insulin	LEC culture
DM15-14	60	M	NIDDM	25	Pancreatic cancer	Unknown	LEC culture
DM16-27	71	F	NIDDM	15	Cardiac	Insulin	LEC culture
DM16-28	88	F	NIDDM	40	Cardiac arrest	Oral DM meds	LEC culture, DNA methylation
DM17-39	86	F	IDDM	25	Cardiac arrest	Unknown	LEC culture
DM18-17	79	F	NIDDM	10	Cardiac arrest	Unknown	*Ex vivo*
DM18-21	73	F	NIDDM	15	Respiratory failure	DM oral medication for 13 years, insulin for 2 years	*Ex vivo*
DM19-28	79	M	NIDDM	15	Cardiac arrest	Unknown	LEC culture, *ex vivo*
DM19-42	73	M	NIDDM	5	Cardiac arrest	Insulin, Metformin,	Organ culture

DM19-48	66	M	NIDDM	30	CAD	Insulin, Glipizide, Insulin Aspart/Detemir	Organ culture
DM19-50	75	M	NIDDM	10	Cardiac arrest	Unknown	Organ culture
DM20-09	80	M	NIDDM	16	Cardiopulmonary arrest	Lantus	Organ culture
DR21-18	64	F	NIDDM	15	Acute cardiac event	Anti-diabetic meds, insulin	Organ culture
DM21-20	77	M	NIDDM	15	Acute cardiac event	Toujeo, Trulicity, Novolin	Organ culture
DM21-21	86	M	NIDDM	20	Basal cell carcinoma	Unknown	Organ culture
DM21-22	61	F	NIDDM	Unknown	Myocardial infarction	Unknown (Poorly controlled DM)	Organ culture
DM21-30	68	M	NIDDM	20	Renal failure	Unknown	Organ culture
DM21-31	60	M	IDDM	25	Myocardial infarction	Insulin	Organ culture
DM22-02	69	F	IDDM	50	Sepsis	Unknown	Organ culture
DM22-14	74	F	NIDDM	5	Respiratory failure	Insulin Lispro	Organ culture

Abbreviations: N, non-diabetic; DM, diabetic; DR, with documented diabetic retinopathy; IDDM, insulin-dependent diabetes mellitus (type 1); NIDDM, non-insulin-dependent diabetes mellitus (type 2); F, female; M, male; N/A, not applicable.

ESM Table 2. Characterisation of NBC

Parameter	Control	Therapeutic
Structure	PMLA/LLL/OKT-9/Control AON/ Alexa488	PMLA/LLL/OKT-9/miR-203 AON/ Alexa488
Polymer molecular mass	85 kDa	85 kDa
AlexaFluor, μmol/l	20.83	19.4
a-TfR OKT-9 mAb, %	0.2%	0.2%
AON, μmol/l	100	90
ζ-potential (mV)	-11.8 ± 0.2	-12.7 ± 0.9

Abbreviations: PMLA, polymalic acid; AON, antisense oligonucleotide; TfR, transferrin receptor; mAb, monoclonal antibody.

ESM Table 3. List of antibodies used

Antigen	Antibody	Source	Dilution	Application
Active caspase-3	Rabbit pAb	559565, BD Pharmingen (Franklin Lakes, NJ)	1:100	IHC
β-Actin	Mouse mAb	A5316, Sigma-Aldrich (St. Louis, MO)	1:2000	WB
β-Actin	Mouse mAb	A5441, Sigma-Aldrich	1:2000	WB
β-Actin	Rabbit mAb	8457, Cell Signaling Technology (Danvers, MA)	1:1000	WB
5-methylcytosine	Mouse mAb	ab10805, Abcam (Waltham, MA)	1:50	IHC
DNMT1	Rabbit mAb	5032S, Cell Signaling Technology	1:1000	WB
DNMT1	Rabbit pAb	ab19905, Abcam	1:50	IHC
Keratin 15	Mouse mAb	sc-47697, Santa Cruz Biotechnology (Santa Cruz, CA)	1:10, 1:50	IHC
Keratin 15	Rabbit mAb	ab52816, Abcam	1:2000	WB
Keratin 17	Mouse mAb	sc-58726, Santa Cruz Biotechnology	1:3, 1:50	IHC
Ki67	Mouse mAb	sc-101861, Santa Cruz Biotechnology	1:10	IHC
Nidogen-1	Mouse mAb	MAB2570, R&D Systems (Minneapolis, MN)	1:10	IHC
Integrin α3β1	Mouse mAb	MAB1992, EMD Millipore (Billerica, MA)	1:100	IHC
p-Akt (S473)	Rabbit pAb	9271, Cell Signaling Technology	1:50	IHC
p-ERK1/2 (T185/Y187)	Rabbit pAb	ab4819, Abcam	1:50	IHC
p-p38 (T180/Y182)	Rabbit mAb	05-1059, EMD Millipore	1:50	IHC
Wnt-3a	Rabbit pAb	ab28472, Abcam	1:50	IHC
Wnt-3a	Rabbit mAb	703666, Thermo Fisher (Carlsbad, CA)	1:25	IHC
Wnt-5a	Rabbit mAb	ab179824, Abcam	1:500, 1:5000	IHC, WB
Wnt-5a	Mouse mAb	MAB10373, Abnova (Taipei, Taiwan)	1:200	IHC
Wnt-5a	Mouse mAb	H00007474-M04, Abnova	1:50	IHC
Wnt-11	Rabbit pAb	PA5-21712, Thermo Fisher	1:50	IHC
Wnt-11	Rabbit pAb	ab31962, Abcam	1:50	IHC

Abbreviations: mAb, monoclonal antibody; pAb, polyclonal antibody; WB, Western blot; IHC, immunohistochemistry. Primary antibodies were diluted in Tris-buffered saline with 0.1% Tween-20 (for WB) and phosphate-buffered saline (for IHC).

ESM Table 4. Protein expression changes after various treatments in organ-cultured diabetic corneas

Marker changes after Wnt-5a treatment of organ-cultured diabetic corneas					
**Marker**	**DM19-42**	**DM19-48**	**DM19-50**	**DM20-09**	**Number of independent cases with expression change *vs.* control**
K15	+	+	+	+	4/4
K17	+	+	+	+	4/4
Integrin α3β1	+	+	+	+	4/4
Nidogen-1	+	+	+	+	4/4
**Marker changes after zebularine treatment of organ-cultured diabetic corneas**					
**Marker**	**DM21-18**	**DM21-20**	**DM22-21**	**DM22-14**	**Number of independent cases with expression change *vs.* control**
DNMT1	+	n/a	+	+	3/3
Wnt-5a	+	n/a	+	+	3/3
K15	+	n/a	+	+	3/3
K17	+	n/a	+	+	3/3
Integrin α3β1	+	n/a	-	+	2/3
Nidogen-1	+	n/a	+	+	3/3
**Marker changes after NBC treatment of organ-cultured diabetic corneas**					
**Marker**	**DM21-22**	**DM21-30**	**DM21-31**	**DM22-02**	**Number of independent cases with expression change *vs.* control**
Wnt-5a	+	+	+	+	4/4
K15	+	+	-	+	3/4
K17	+	+	-	+	3/4
Integrin α3β1	+	+	-	+	3/4
Nidogen-1	+	-	+	+	3/4

+, increased expression; -, no change.

ESM Table 5. Wnt-5a dependent activation/phosphorylation of wound healing mediators in non-diabetic and diabetic LEC

**Wound healing mediator**	**Non-diabetic**		**Diabetic**	
	**Control**	**Wnt-5a**	**Control**	**Wnt-5a**
p-Akt (S124)/Akt	1	0.89	1	1.63
p-Akt (S473)/Akt	1	0.76	1	1.21
p-ERK3 (S189)/ERK3	1	0.70	1	1.30
p-ERK8 (T175/Y177)/ERK8	1	0.75	1	1.72
p-TAK1 (S439)/TAK1	1	1.03	1	1.97
p-Src (S75)/Src	1	0.96	1	1.32

Data from Wnt phospho arrays are shown. Treatment was with 200 ng/mL recombinant Wnt-5a. Numbers are fold changes of activated signaling mediator *vs.* respective total mediator.

a b

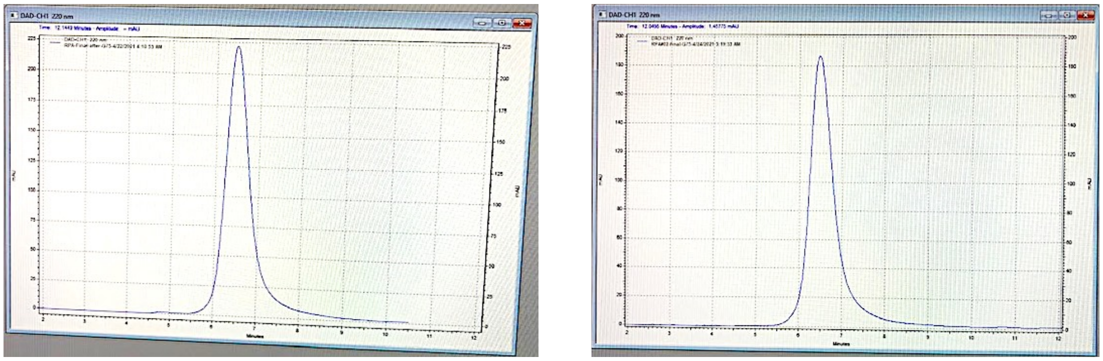

PMLA/LLL/OKT-9/Control AON/Alexa488

PMLA/LLL/OKT-9/miR203 AON/Alexa488

ESM Figure 1. SEC-HPLC analysis of nanoconjugates. (a) PMLA/LLL/OKT-9/Control AON/Alexa488 and (b) PMLA/LLL/OKT-9/miR203 AON/Alexa488. HPLC pump: Hitachi L-2130; detector, Hitachi L-2455; software, EZChrome; Column, Polysep 4000; flow rate: 1mL/min; buffer, PBS.

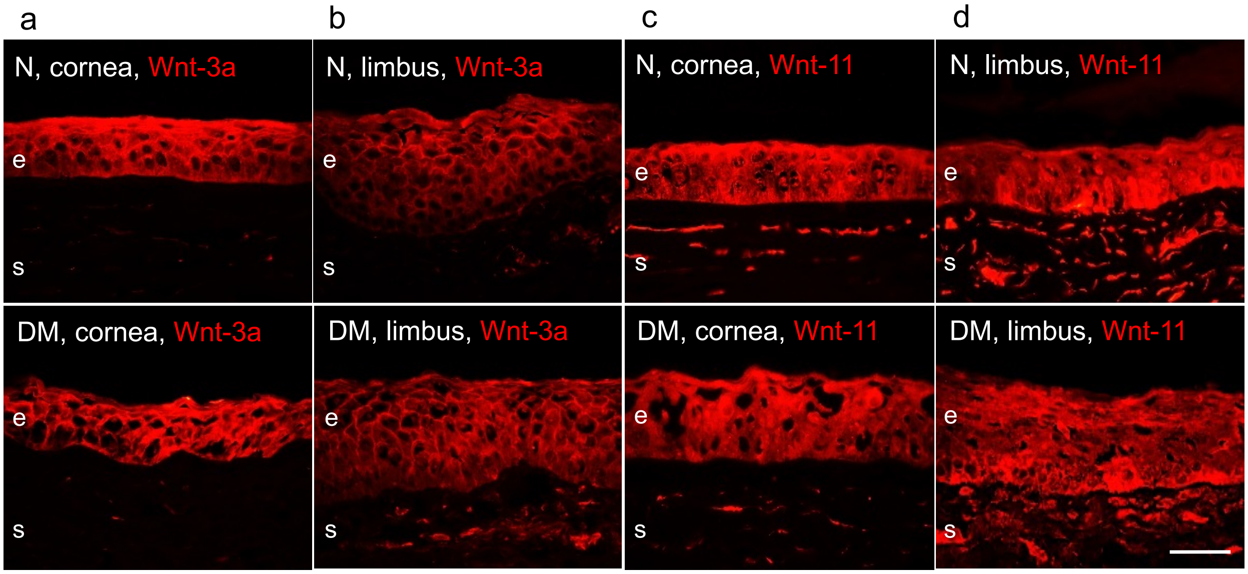

ESM Figure 2. Immunostaining of canonical Wnt-3a and non-canonical Wnt-11 in *ex vivo* human non-diabetic and diabetic corneal sections. Wnt-3a in (a) central cornea and (b) limbus, and Wnt-11 in (c) central cornea and (d) limbus in *ex vivo* non-diabetic and diabetic sections. Both components show no change in diabetic corneas. N, non-diabetic; DM, diabetic. e, epithelium; s, stroma. Scale bar, 20 μm.

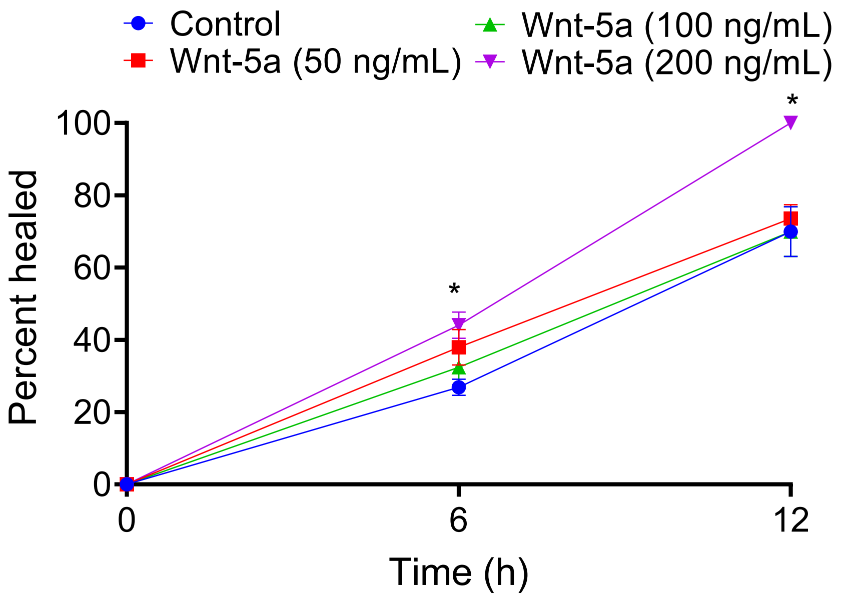

ESM Figure 3. Dose-dependent stimulation of scratch wound healing in diabetic LEC by recombinant human Wnt-5a. Values are mean ± *SEM*. *, $p<0.05$ *vs*. control at the same time point. Paired *t*-test. Number of independent cases n=3.

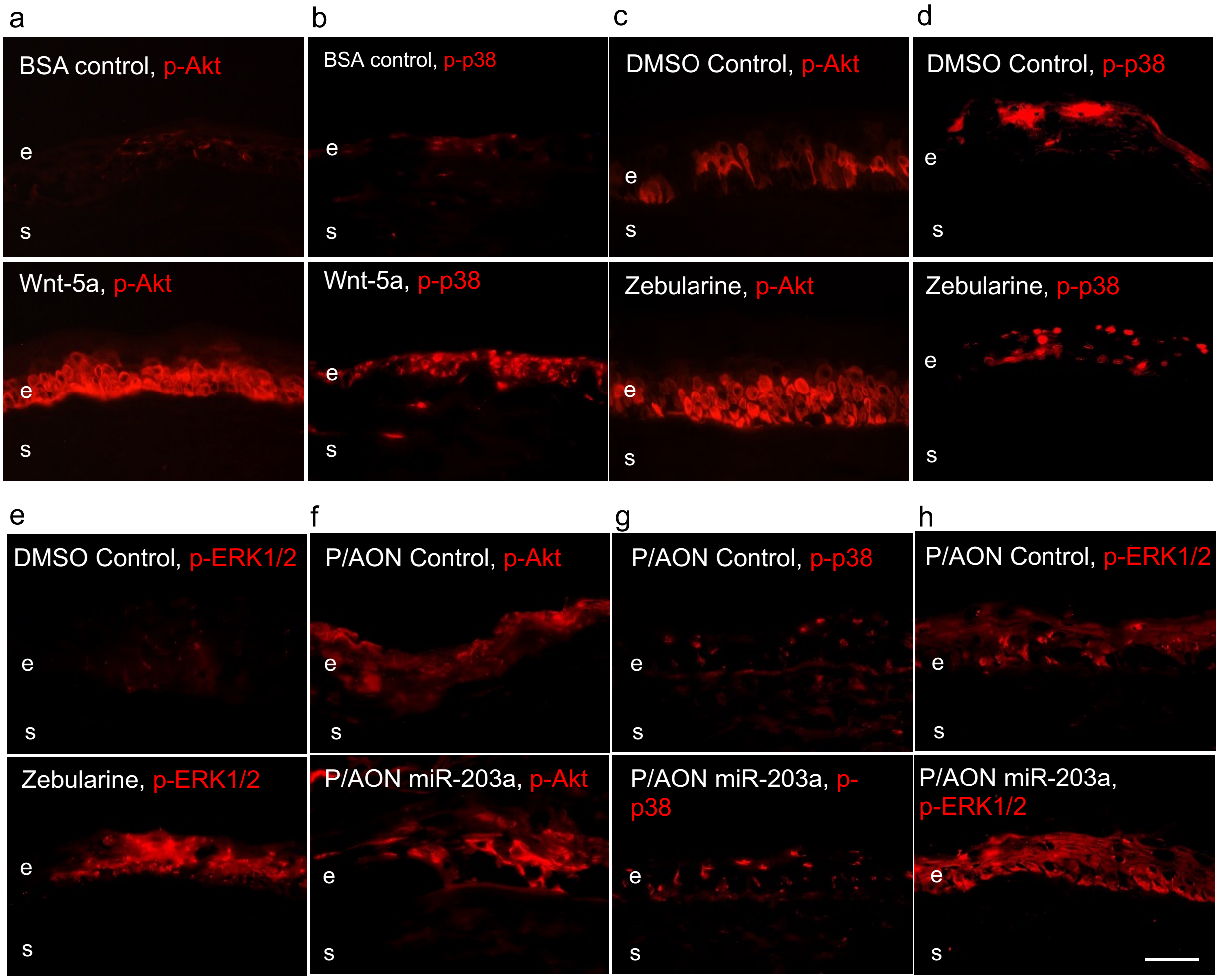

ESM Figure 4. Immunostaining for activated signaling intermediates p-Akt (S473), p-p38 (T180/Y182), and p-ERK1/2 (T185/Y187) in diabetic organ-cultured corneas. Corneas were treated with Wnt-5a (200 ng/mL) or BSA control (a, b); zebularine (20 μmol/l) or DMSO control (c, d, e); P/AON miR-203a (20 μmol/l) or P/AON control (f, g, h). The levels of signaling components, especially, of p-Akt, were increased upon various treatments. e, epithelium; s, stroma. Scale bar, 20 μm.

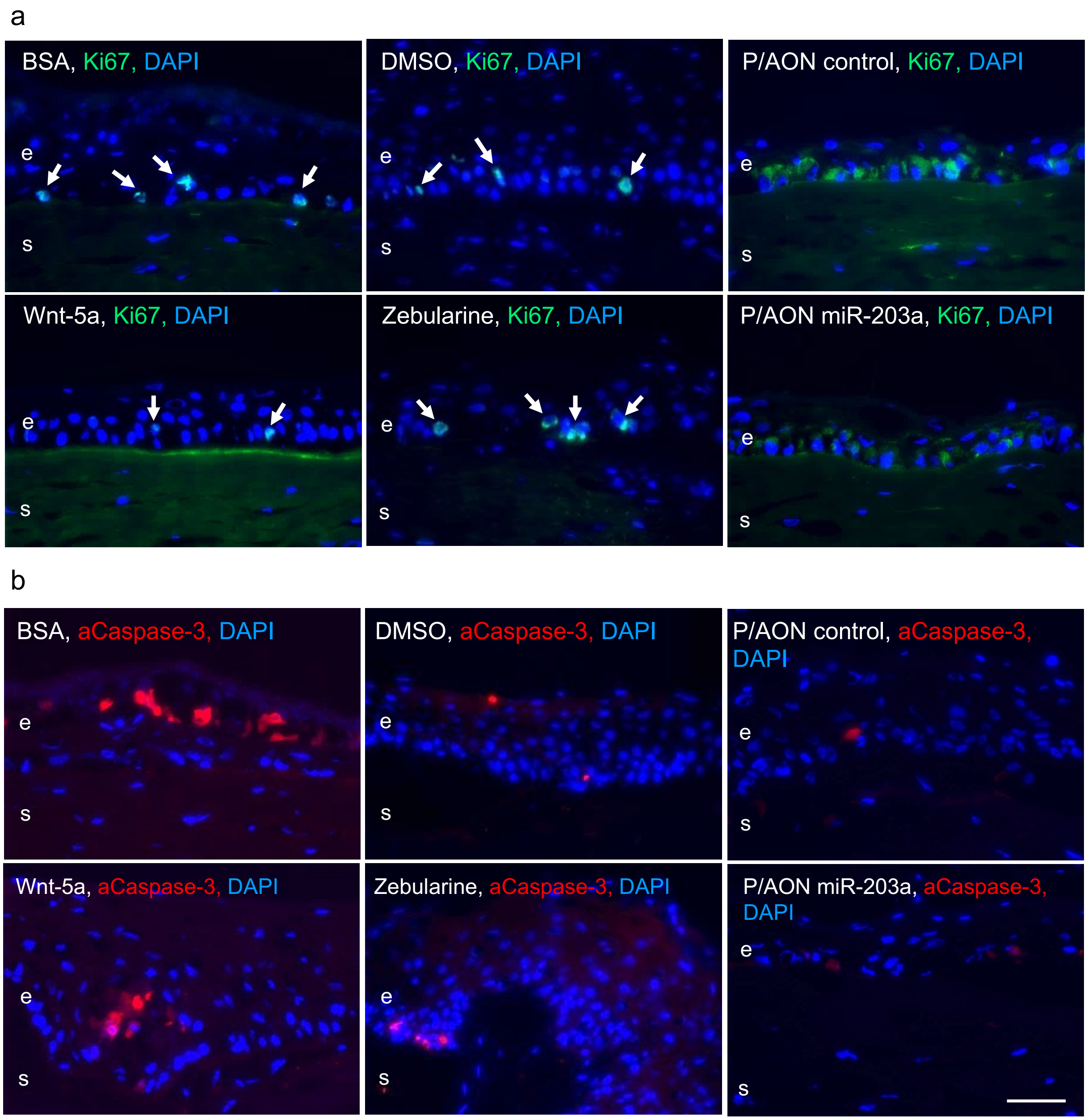

ESM Figure 5. Proliferation and apoptosis in Wnt-5a-, zebularine-, or NBC-treated diabetic organ-cultured corneas. Immunostaining of diabetic organ-cultured corneas for nuclear proliferation marker Ki67 (a) or apoptosis marker activated caspase-3 (b) did not reveal any effects of Wnt-5a, zebularine, or NBC *vs.* respective controls. BSA and Wnt-5a were used at 200 ng/mL, DMSO and zebularine, at 20 μmol/l, P/AON control and P/AON miR-203a, at 20 μmol/l AON. e, epithelium; s, stroma. Scale bar, 20 μm.

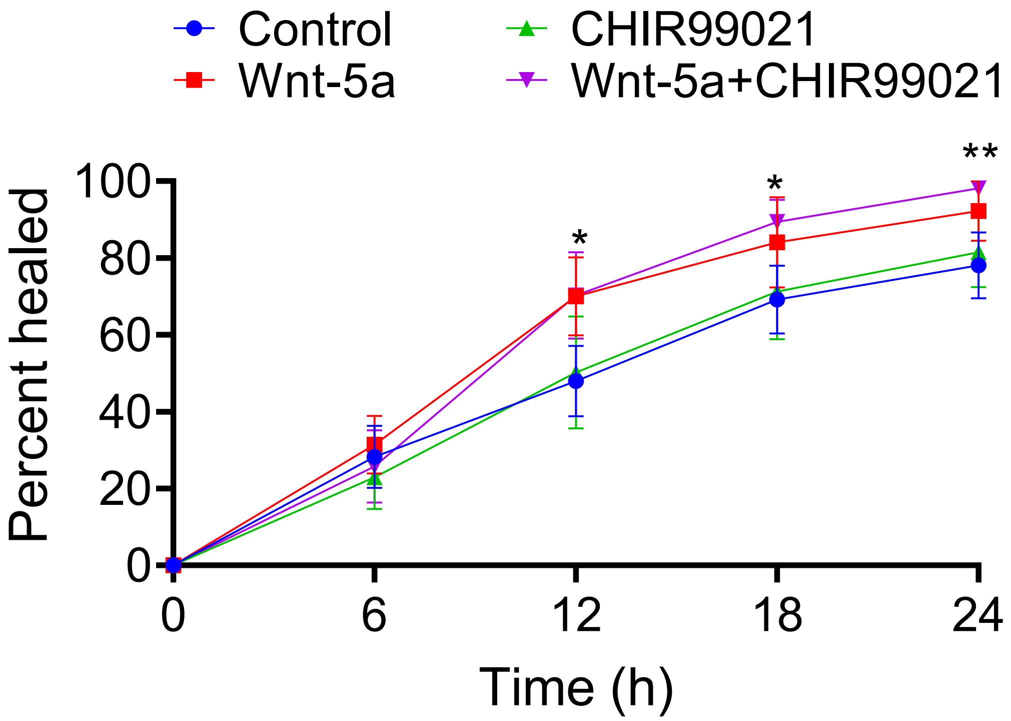

ESM Figure 6. Wound healing of diabetic LEC with or without Wnt-5a and/or GSK3β inhibitor CHIR99021. Wnt-5a was used at 200 ng/mL and GSK3β inhibitor (canonical Wnt signaling activator) at 5 μmol/l. The inhibitor did not have a significant effect, suggesting minor involvement of canonical Wnt signaling in Wnt-5a effects and baseline wound healing. Values are mean ± *SEM*. *, $p<0.05$; ** $p<0.01$ *vs*. control at the same time point. Paired *t*-test. Number of independent cases n=3.

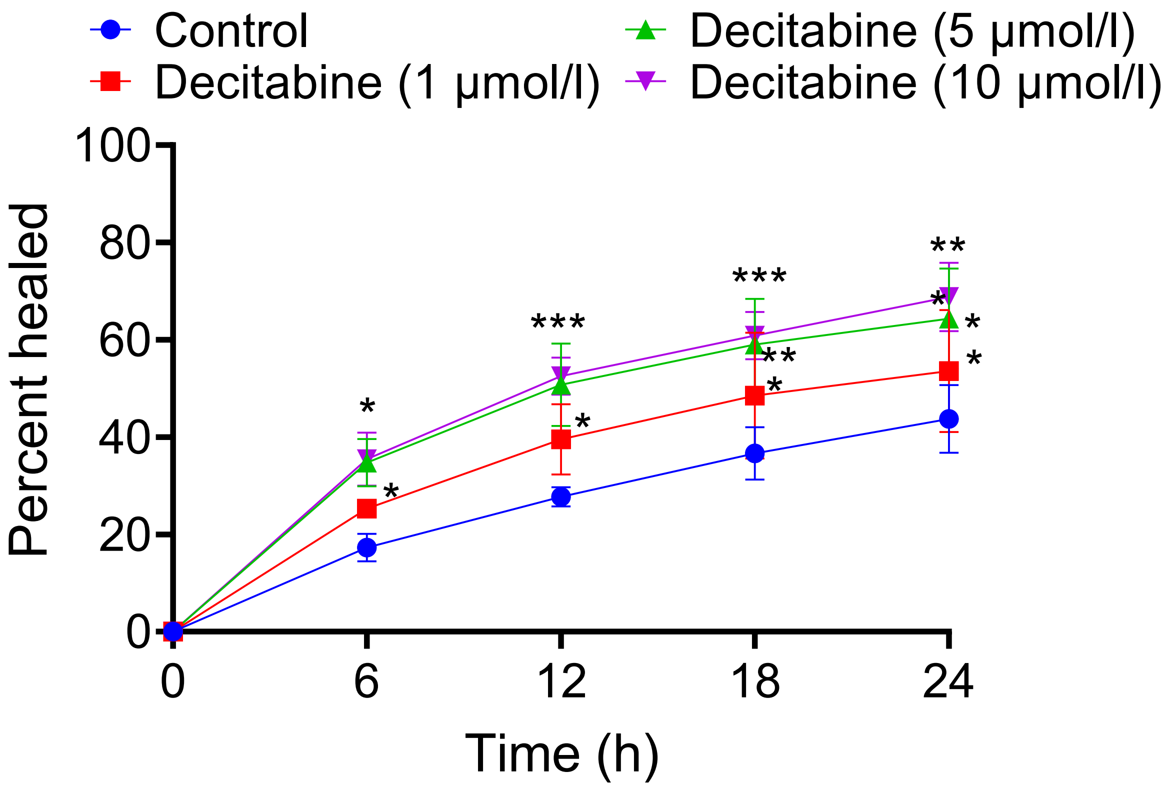

ESM Figure 7. Dose-dependent stimulation of scratch wound healing in diabetic LEC by Decitabine. Values are mean ± *SEM*. *, p<0.05; **, p<0.01; ***, p<0.001 *vs*. control at the same time point. Paired *t*-test. Number of independent cases n=3.

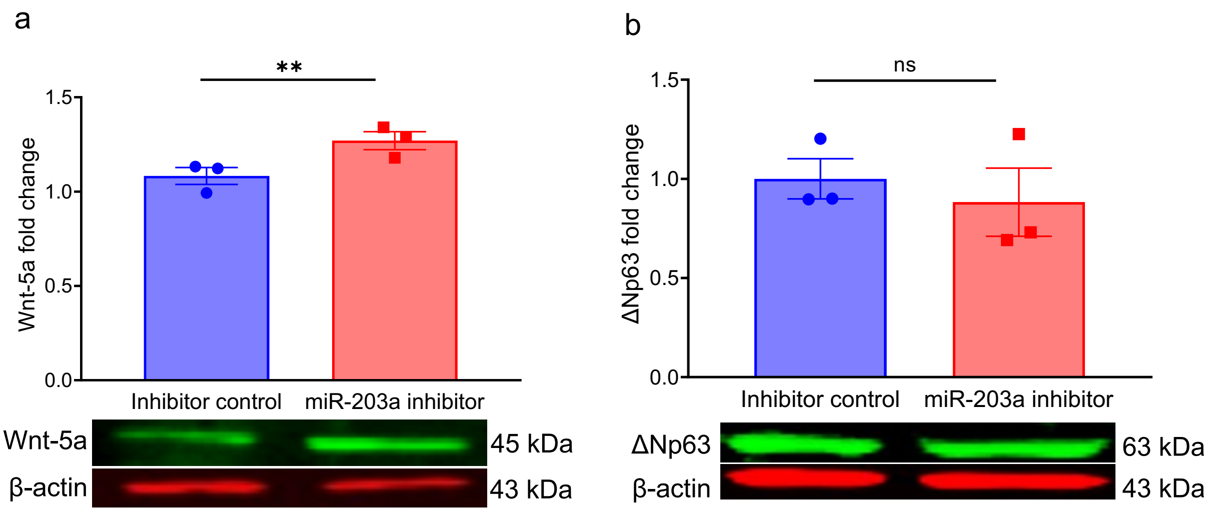

ESM Figure 8. Protein levels of Wnt-5a and ΔNp63 in diabetic LEC transfected with miR-203a inhibitor and inhibitor control. (a) Wnt-5a and (b) ΔNp63 proteins in diabetic LEC transfected with miR-203a inhibitor and inhibitor control. miR-203a inhibitor caused increase of Wnt-5a but not of ΔNp63. Values are mean ± *SEM*. *, p=0.05 *vs.* control. Paired t-test. Number of independent cases n=3. Control value is taken as 1.
